# Supplementary material for: The interactome of intact mitochondria by cross-linking mass spectrometry provides evidence for coexisting respiratory supercomplexes
Source: Mol Cell Proteomics. 2017 Dec 8;17(2):216–32. doi: 10.1074/mcp.RA117.000470 (PMC5795388; doi:10.1074/mcp.RA117.000470)
Supplement: Supplemental Data [file supp_RA117.000470_133922_0_supp_23978_fzffwf.zip › spectra_annotation/mito_DR_spectra_annotation/250-1-1-1-1-1.pdf]

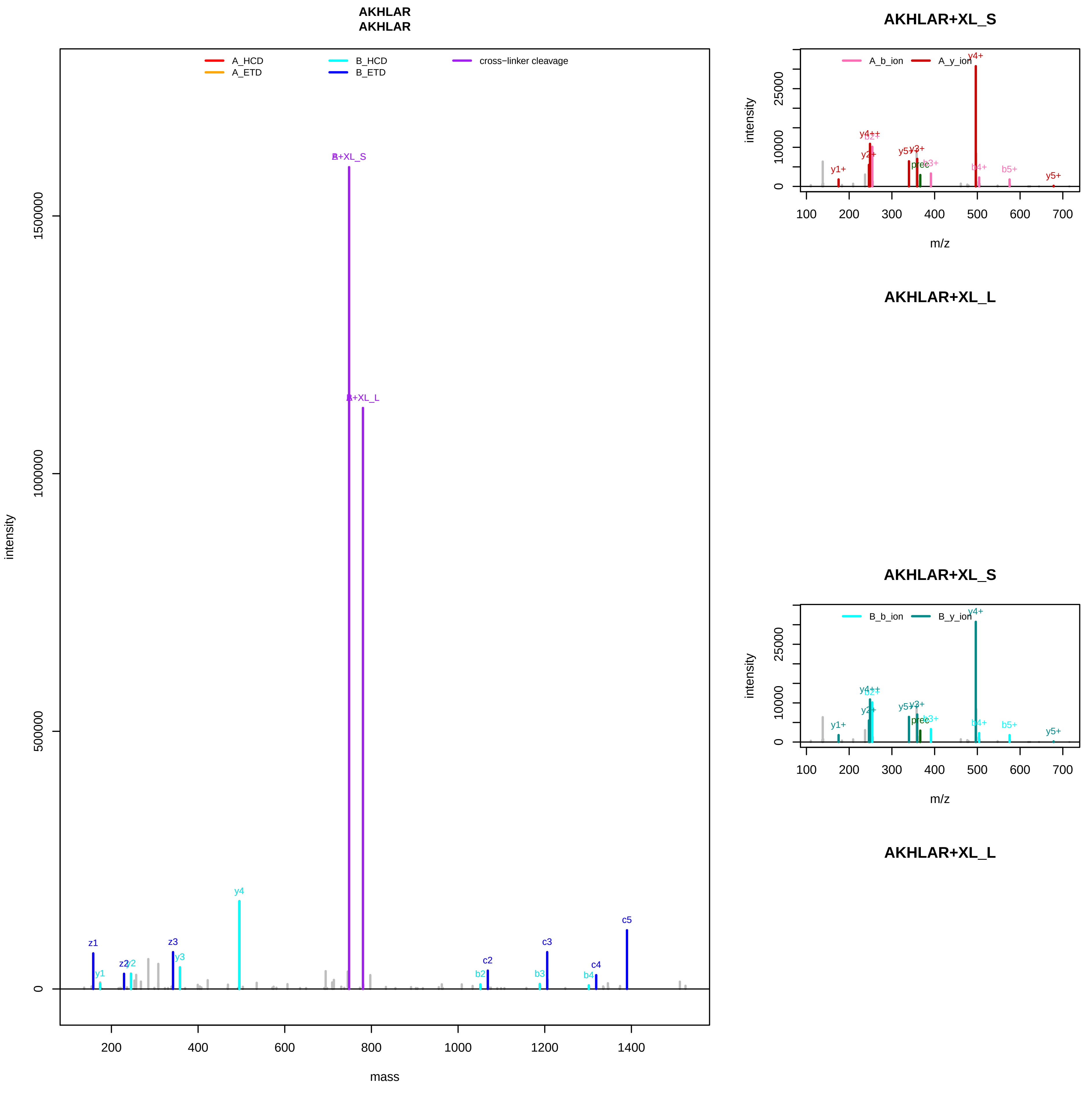

AKHLAR
AKHLAR
A_HCD
A_ETD
B_HCD
B_ETD
cross−linker cleavage
B+XL_S
B+XL_L
intensity
mass
y4
c5
z1
y1
z2
y2
z3
y3
c2
b2
c3
b3
c4
b4
0
500000
1000000
1500000
200
400
600
800
1000
1200
1400
AKHLAR+XL_S
A_b_ion
A_y_ion
y4+
y4++
b2+
y2+
y5++
y3+
prec
b3+
y1+
b4+
b5+
y5+
intensity
m/z
0
10000
25000
100
200
300
400
500
600
700
AKHLAR+XL_L
AKHLAR+XL_S
B_b_ion
B_y_ion
y4+
y4++
b2+
y2+
y5++
y3+
prec
b3+
y1+
b4+
b5+
y5+
intensity
m/z
0
10000
25000
100
200
300
400
500
600
700
AKHLAR+XL_L
